# Association between antibodies to *Coxiella burnetii *in bulk tank milk and perinatal mortality of Danish dairy calves

**DOI:** 10.1186/1751-0147-53-64

**Published:** 2011-12-02

**Authors:** Katrine T Nielsen, Søren S Nielsen, Jens F Agger, Anna-Bodil Christoffersen, Jørgen S Agerholm

**Affiliations:** 1 Department of Large Animal Sciences, Faculty of Life Sciences, University of Copenhagen, Grønnegårdsvej 8, DK-1870 Frederiksberg C, Denmark; 2 National Veterinary Institute, Technical University of Denmark, Bülowsvej 27, DK-1790 Copenhagen V, Denmark

**Keywords:** Cattle, *Coxiella burnetii*, perinatal death, stillbirth

## Abstract

**Background:**

*Coxiella burnetii *is a well-known cause of placentitis and subsequent abortion in ruminants, but there are no reports on the relationship with perinatal mortality. The study was performed to determine the influence of level and change of bulk tank milk (BTM) antibodies to *C. burnetii *on two outcomes associated with parturition in cattle: a) stillbirth; and b) stillbirth and neonatal mortality combined (perinatal death).

**Methods:**

Twenty-four Danish dairy herds were tested repeatedly for antibodies to *C. burnetii *in BTM using a commercial ELISA. Samples were collected monthly from July 2008 to July 2009. Information on the 2,362 calvings occurring in the study period was obtained from the Danish Cattle Database. Two multilevel logistic regression models were created for the two outcomes stillbirth and perinatal mortality. One model included the level of BTM antibodies in a specified period before or after the outcome had occurred. The other model included the change in antibodies over time. These predictors were included both at herd and animal level. Furthermore, all models included parity and breed.

**Results:**

The individual monthly BTM antibody levels were highly correlated within herds. Consequently, changes in BTM antibody levels were not found to be associated with neither risk of stillbirth nor the risk of perinatal mortality. However, the risk of stillborn calves and perinatal death was higher with high level of BTM antibodies 8 to 9 months after the incident, but not outside this period.

**Conclusion:**

We conclude that the level of antibodies to *C. burnetii *in BTM may be associated with perinatal mortality, but the association was not persistent and should be investigated further.

## Background

*Coxiella burnetii *is a cause of sporadic abortion in cattle [1,2]. The infection occurs almost world-wide and recent studies of bulk tank milk (BTM) antibodies in some European countries have shown between-herd prevalences ranging from 38 to 79% in cattle [3-5]. *C. burnetii *has been detected in the vagina of cattle [6], and recently infection with *C. burnetii *without associated placental pathology was reported [7]. *C. burnetii *associated abortions in ruminants are characterized by extensive necrotizing placentitis [2]. However, there is no reason to believe that *C. burnetii *associated placentitis differs from other bacterial infections of the pregnant uterus, where foetuses with the most severe infection are aborted or delivered prematurely, while less severe infections result in stillbirth, delivery of weak offspring, congenital infections or even normal offspring. This is a feature of coxiellosis (Q fever) in pregnant women [8].

The aim of this study was to evaluate if BTM *C. burnetii *antibody level is associated with stillbirth and perinatal calf mortality.

## Materials and methods

### Study design and sample selection

**Figure 1:**
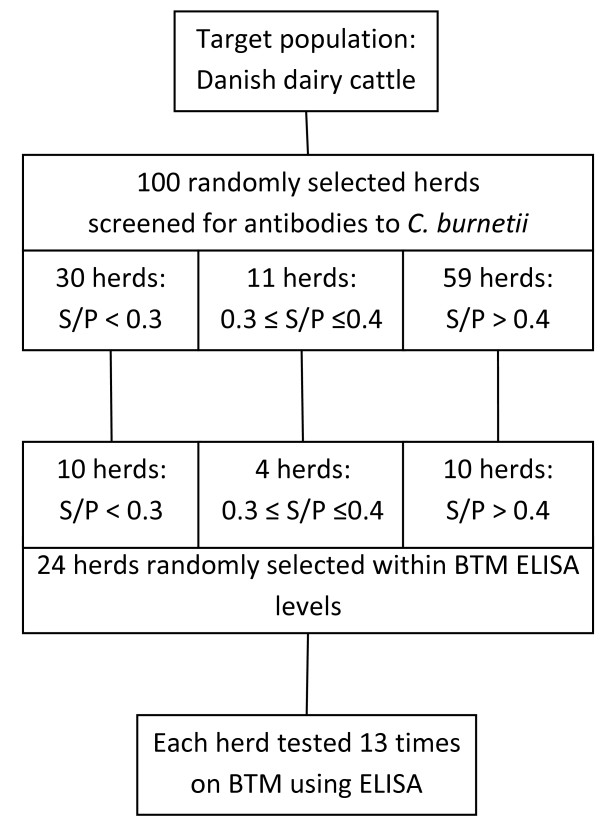
**Outline of study design**. Stratified selection of 24 herds from a random sample of 100 herds.

One hundred randomly selected dairy herds were previously screened for antibodies to *C. burnetii *in BTM [3]. These herds were divided into three categories depending on the levels of BTM antibodies. A stratified random selection of 10, 4 and 10 herds within groups of high, intermediate and low concentrations of antibodies ensured that all groups were represented in the study (Figure 1). The study population consisted of 14 Danish Holstein herds, 5 Danish Jersey herds, 1 Danish Red herd and 4 herds with mixed breeds. The mean number of lactating cows in the herds was 84 (range 21-254) at the date of the first sampling.

**Figure 2:**
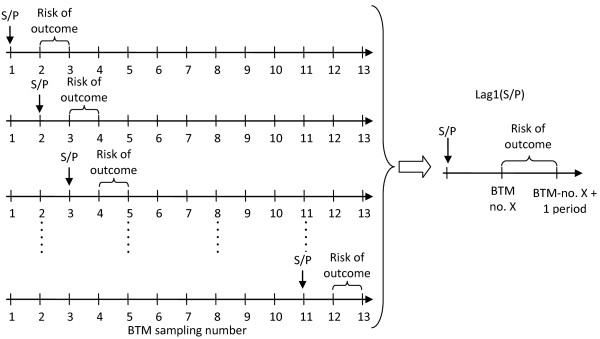
**Analytical strategy**. For each outcome variable, the risk of the outcome was assessed in a period before or after recording of the S/P ratio of *C. burnetii *in BTM. Here, the S/P-value measured one period before the outcome is shown. S/P-values could thus be up to 12 periods before and 12 periods after occurrence of the outcome.

The study was organised as a longitudinal study, where the outcome variables (see below) were assessed relative to BTM recordings retrospectively, cross-sectionally or prospectively (Figure 2).

#### Data collection

BTM samples were submitted by farmers by postal services monthly from July 2008 to July 2009. Each herd contributed 13 samples, except for one herd exiting the study after 4 tests because the farmer became sick. Data on the calvings were recorded continuously by the farmer as part of the routine herd management and stored in the Danish Cattle Database according to Danish legislation. These data together with breed and parity information were obtained retrospectively for the same period as the BTM samples. Cattle were grouped into Danish Holsteins (n = 1,955), Danish Jerseys (n = 767), and others (n = 305). The latter group consisted of 191 Danish Red and 114 crossbreds. Three parity groups were created: parity 1, parity 2 and parity > 2.

#### BTM serology

BTM samples were sent to the laboratory, centrifuged and tested for antibodies to *C. burnetii *using a commercial ELISA (CHEKIT ELISA, IDEXX, Liebefeld-Bern, Switzerland) according to instructions of the manufacturer. The test is based on inactivated *C. burnetii *antigens from both phase 1 and phase 2 reactions. The result of the ELISA is reported as a sample to positive ratio, calculated from the optical density (OD) of the sample, a positive and a negative control: ((OD_Sample_- OD_Negative control_)/(OD_Positive control_- OD_Negative control_). The S/P-ratio represents the level of antibodies in the BTM sample.

#### Outcome parameters

Two outcome parameters were considered based on the routinely collected data: a) "stillbirth", defined as a stillborn calf born later than 270 days after last insemination; and b) "perinatal death" including "stillbirth" and calves dying within 24 hours post partum. Only 14 calves from 2,362 calvings were recorded as dying within 24 hours after birth. Consequently, death within 24 hours could not be used as an outcome alone. Recording of stillbirths and post natal mortality in cattle has a very long tradition in the Danish dairy industry. It is mandatory and is done systematically. The recording is done by the breeder and reported to the Danish Cattle Database. As the parameter (dead *vs*. alive) is obvious to the breeder, the data are considered of high quality.

#### Statistical methods

##### Analytical strategy

Cross-sectional studies do not capture infection dynamics within herds, whereas the present longitudinal study design could reveal dynamics in herd-level immune response over a one year period. First the two outcomes were studied without considering dynamics by assessing the S/P-level (LEVEL analysis). Then the increase in S/P-ratios between two sampling dates was evaluated in a similar way to include such dynamics (INCREASE-analysis). This study included analyses assessing perinatal calf mortality both before and after the estimated level of BTM antibodies. The rationale behind looking forward was that high levels of antibodies has been associated with high levels of *C. burnetii *in milk at least 6 months prior to occurrence of antibodies [9]. Thus, the effect of *C. burnetii *on perinatal mortality would be expected to have occurred in the past. However, if single early infections lead to high BTM antibody levels early in an epidemic, and the majority of cows are only affected in a subsequent parity, it might be more reasonable to look backward. The risk of each outcome was estimated for each interval between consecutive BTM test-dates. This risk was defined as the proportion of the outcome among all calvings occurring in the sample interval. The risk was then associated with the S/P-ratio in each of the previous, concurrent and following intervals. For example, the outcome risk in one interval associated with the S/P-ratio on the last sample date prior to the outcome interval is shown in Figure 2 (with further illustration in Additional file 1, Figure S1). This procedure was carried out two sample dates back, three sample dates back etc. The same was done for sampling dates occurring after the outcome interval. Each S/P-ratio was thus associated with 25 outcome intervals, 12 test-dates prior to the outcome interval, 1 in the outcome interval and 12 after the outcome interval. These S/P-values were used to assess the association of the level of the S/P-ratio on the occurrence of the outcome (LEVEL-analysis).

For the INCREASE-analysis, the four parameters Increase01, Increase03, Increase06 and Increase12 were created to represent increases in 1-month, 3-months, 6-months and 12-months intervals, respectively.

##### Descriptive statistics

Spearman's correlation coefficients were estimated to determine the correlation between the repeated S/P ratios within herds. Histograms of S/P-ratios in the risk interval starting on the test-date were created to inspect the distributions for each of stratum of each outcome. Similarly, histograms were created for the increases in S/P-ratios associated with each stratum of each outcome.

##### Analytical statistics

Two statistical models were created to assess the effect of the S/P-ratios on the two outcomes. One model was used to assess the impact of the level of S/P-ratios at a specific time relative to the outcome risk (LEVEL model) and one was used to assess the impact of increases in S/P-ratios in a specific interval relative to the outcome risk (INCREASE model). Both models included the three breeds and three parity groups, as they were expected to influence the outcome risks. Because S/P-ratios were recorded on herd level, and outcomes, parity group and breed group on cow-level, a two-level random coefficient logistic regression model was created with the following general structure:

$$\text{logit}\left(\pi \right)=\text{A}+\text{B}+\text{P}+\text{b}\times \text{SP}+\mu +\gamma \times \text{SP},$$

where π is the odds of a cow having the outcome of interest, A is the baseline probability of having the outcome, B is the effect of breed group (Danish Holstein, Danish Jersey or other), P is the effect of parity group (1, 2 or > 2), SP is the effect of level or increase of S/P ratio (for the LEVEL and INCREASE models, respectively), with b being the cow-level coefficient and γ the herd-level coefficient, and μ is the random intercept for herd. All two- and three-way interactions between cow-level factors were assessed. Each LEVEL model was run for each time specific S/P ratio before, during and after the outcome interval. Each INCREASE model was done for changes over 1, 3, 6 and 12 months relative to the outcome interval.

The level of significance was set to 5%.

The model fit was assessed by a) convergence of the model; and b) the extra dispersion scale. If the model did not converge or the extra dispersion scale was < 0.95 or > 1.05, the model was run including only Danish Holsteins, which was the largest herd group. All analyses were done using the Glimmix macro in SAS version 9.2 (SAS Institute, Cary, NC, USA).

## Results

### Descriptive statistics

The correlation between BTM antibody recordings are shown in Table 1. Correlations between samples taken 1 month apart were in the range 0.85 to 0.93. The minimum correlation between samples obtained up to 12 months apart was 0.67.

**Table 1:** Within-herd Spearman's correlation coefficients for repeated sample-to-positive ratios of *Coxiella burnetii *bulk tank milk antibody recordings in 24 Danish dairy herds.

	N	Lag1	Lag2	Lag3	Lag4	Lag5	Lag6	Lag7	Lag8	Lag9	Lag10	Lag11	Lag12
S/P	290	0.87	0.85	0.82	0.78	0.79	0.80	0.78	0.75	0.74	0.73	0.74	0.78
Lag1	266	1	0.87	0.85	0.81	0.76	0.78	0.78	0.76	0.75	0.70	0.70	0.80
Lag2	242		1	0.87	0.84	0.79	0.75	0.77	0.75	0.75	0.71	0.68	0.87
Lag3	219			1	0.87	0.84	0.79	0.73	0.76	0.75	0.72	0.70	0.73
Lag4	196				1	0.86	0.83	0.77	0.72	0.75	0.72	0.70	0.78
Lag5	173					1	0.86	0.82	0.76	0.73	0.75	0.67	0.70
Lag6	150						1	0.86	0.81	0.75	0.70	0.75	0.72
Lag7	127							1	0.87	0.82	0.74	0.68	0.92
Lag8	104								1	0.86	0.79	0.76	0.86
Lag9	81									1	0.85	0.81	0.92
Lag10	58										1	0.87	0.78
Lag11	35											1	0.92

N is the number of samples included in the estimation. Lag1 expresses the previous recording; Lag2 is two recordings earlier etc.

**Figure 3:**
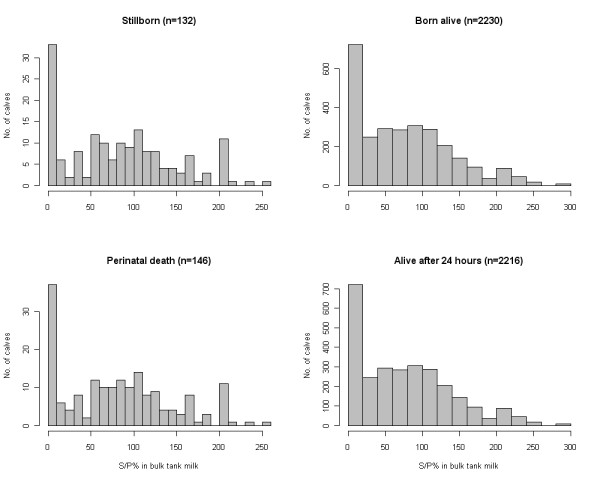
**Distribution of S/P values for the 2 outcomes stratified by cases and non-cases**.

**Figure 4:**
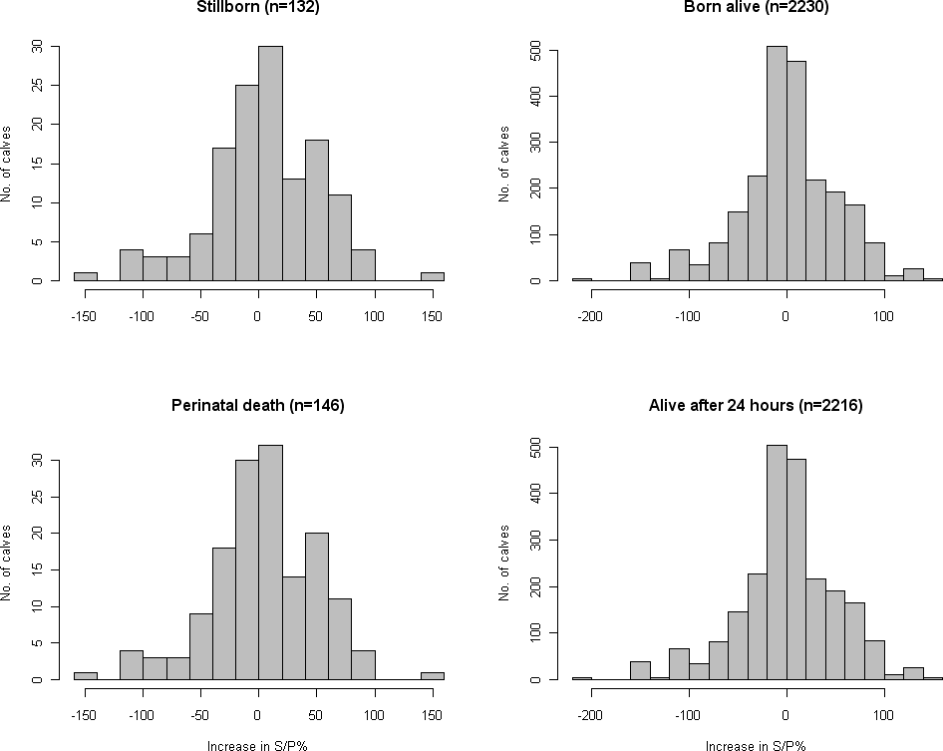
**Distribution of increases in S/P values for the stillbirth (top) and perinatal mortality bottom**.

A total of 2,362 calvings were included in the analyses. Of these, 132 resulted in a stillbirth and 14 calves died within 24 hours post partum resulting in 146 calves experiencing perinatal death, i.e. 5.6%. The distributions of S/P ratio levels and S/P ratio increases associated with each outcome and outcome stratum are shown in Figures 3 and 4, respectively.

### Analytical statistics

All models converged and had sufficient model fit. No significant interactions were detected in any of the models, and there was no effect of breed group. However, parity group was persistently significant and was thus retained in all models.

**Figure 5:**
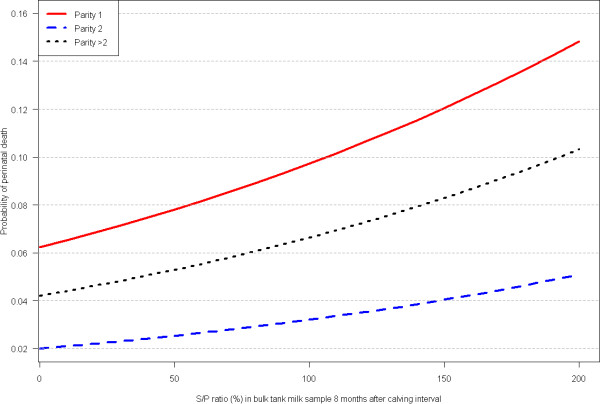
**Result from the logistic analysis on perinatal death**. The association between S/P level 8 months after calving and the probability of perinatal death.

In the LEVEL model, the risk of stillbirth was not associated with the S/P-level. Increased perinatal death was associated with high S/P values occurring 8 months after the incident (Table 2). The modelled effect is illustrated in Figure 5 for the S/P level 8 months after the stillbirths.

**Table 2:** Results of the effect of *Coxiella burnetii *bulk tank milk antibody level measured before and after the occurrence of stillborn calves and perinatal death (including stillbirths) of calves in 24 Danish dairy herds.

Time difference (months) between S/P- test date and outcome interval^#^	n(total)	Outcome					
		
		Stillborn			Dead within 24 hours		
		
		n(cases)	Risk	*P*-value	n(cases)	Risk	*P*-value
-12	107	10	0.093	0.20	11	0.103	0.19
-11	250	17	0.068	0.88	18	0.072	0.76
-10	425	29	0.068	0.47	31	0.073	0.27
-9	546	34	0.062	0.44	37	0.068	0.26
-8	750	49	0.065	0.22	55	0.073	0.12
-7	907	57	0.063	0.12	65	0.072	0.07
-6	1065	71	0.067	0.39	80	0.075	0.34
-5	1266	83	0.066	0.16	94	0.074	0.11
-4	1472	90	0.061	0.85	103	0.070	0.51
-3	1725	98	0.057	0.46	112	0.065	0.58
-2	1949	107	0.055	0.23	121	0.062	0.38
-1	2196	120	0.055	0.45	134	0.061	0.73
0	2362	132	0.056	0.23	146	0.062	0.41
1	2098	114	0.054	0.27	127	0.061	0.49
2	1936	102	0.053	0.49	115	0.059	0.78
3	1618	91	0.056	0.32	104	0.064	0.49
4	1411	82	0.058	0.85	95	0.067	0.91
5	1173	74	0.063	0.91	86	0.073	0.59
6	1064	67	0.063	0.15	77	0.072	0.10
7	867	56	0.065	0.24	64	0.074	0.19
8	663	43	0.065	0.04	50	0.075	0.02
9	565	36	0.064	0.17	41	0.073	0.07
10	370	21	0.057	0.46	23	0.062	0.29
11	252	16	0.063	0.30	17	0.067	0.24
12	81	4	0.049	0.65	4	0.049	0.65

#: Negative values for bulk tank milk test dates occurring before the outcome interval.

Abbreviations: S/P: sample-to-positive ratio in *Coxiella burnetii *bulk tank milk antibody ELISA.

## Discussion

This is the first study investigating associations between *C. burnetii *antibody levels and perinatal death in cattle herds. The association was assessed as concurrent measures, as outcomes occurring later that the BMT measure, and as the "outcome" occurring prior to the BTM measure. The rationale behind this strategy was that the pathogenesis and time relationship between BTM S/P levels and stillbirth and perinatal calf mortality is still not known, i.e. does BTM S/P raise before or after or concurrent with stillbirth and perinatal death. The risk of perinatal death was two-fold higher in herds with very high levels of BTM antibodies compared to those with no antibodies (Figure 5), but only when the antibody level was high 8 to 9 months after parturition. A more persistent effect could have been expected, because all our INCREASE analyses were non-significant and the between sample correlation was very high. Although not statistically significant, increases in BTM antibody levels seemed to be associated with increased risk of perinatal death (data not shown). This suggests that the statistical power was insufficient rather than indicating a lack of effect. But also that effects of *C. burnetii *infection on perinatal deaths is limited and associated with some variation similar to human coxiellosis [8] if present at all. However, BTM analyses are herd samples and the association between individual animal antibody levels and calving results were not included. Such analysis may reveal associations.

The three modelled slopes representing the three parity groups (Figure 5) are comparable, but baseline values (S/P ratio = 0) differ with parity = 1 having the highest baseline risk. The higher baseline risk in the parity = 1 group reflects a higher risk of dystocia in 1^st ^parity cows and supports the validity of our model. The reason that parity > 2 baseline has higher risk than parity = 2 is that the parity > 2 group includes the oldest cows with high risk of dystocia and associated risk of perinatal death [10].

The study depended on specific antibody recordings in BTM. *C. burnetii *antibodies can be excreted in milk, but it is uncertain if all infected animals excrete antibodies and if so, at which level. *C. burnetii *phase II antibodies have been associated with occurrence of *C. burnetii *in placentas, whereas phase I antibodies have not [11]. Moreover, sero-negative animals can harbour *C. burnetii *in the placenta [7,11]. The finding of an association between herd level antibodies occurring 8 to 9 month after calving questions the pathogenesis. The correlation between infection and antibody excretion is still poorly understood. A possible explanation may be that detectable antibodies are only present in the lactation following the calving where infection occurred, whereas the calving coinciding with infection results in higher perinatal mortality but with delayed production of antibodies. However, this hypothesis is at present highly speculative. Although not significant it should be mentioned that results in Table 2 show a borderline significant association (p = 0.07) between perinatal death and increased antibody level 7 months later. Presence of antibodies may just indicate past exposure to *C. burnetii *in the herd and therefore not the actual bacterial load at actual pregnancy and parturition. The specific state of infection in the herd or in individual animals could thus not be assessed while using antibodies in BTM. Furthermore, as not all animals in a herd are infected, the BTM level would be affected by the within-herd prevalence and by the individual milk yield, since BTM antibody level is an aggregated score for the entire herd. A recent German study suggested that among the majority of infected herds, the within-herd prevalences were below 10% [11]. This indicates that many herds have antibodies in BTM, but only few animals contribute to the status. Thus, results based on BTM measurements might be misinterpreted because antibodies in the BTM most likely are produced by a few animals in the herd, and perinatal mortality has other causes than coxiellosis as well. BTM data most likely underestimate the effect or leave it unnoticed. Therefore, to elucidate the pathogenesis, it would be necessary to test individual animals for further knowledge about the influence of infection status on stillbirth and perinatal death.

## Conclusions

A clear association between BTM level of antibodies against *C. burnetii *and perinatal mortality was not found, but the risk of perinatal death was two-fold higher in herds with very high levels of BTM antibodies 8 to 9 months after parturition compared to those with no antibodies However, the mechanism behind cannot be explained and requires further scrutiny.

## Competing interests

The authors declare that they have no competing interests.

## Authors' contributions

JFA, ABC and JSA designed the sampling protocol and collected the samples. ABC was responsible for the laboratory analyses. KTN and SSN designed and performed the epidemiological and statistical analyses. KTN, SSN and JSA drafted the manuscript, and all authors contributed to, and approved, the final manuscript.

## Supplementary Material

Additional file 1 **Outline of study design**. Illustration of the relationship between S/P-values relative to the occurrence of the outcome. Click here for file
