# Supplementary material for: Association between antibodies to Coxiella burnetii in bulk tank milk and perinatal mortality of Danish dairy calves
Source: Acta Vet Scand. 2011 Dec 2;53(1):64. doi: 10.1186/1751-0147-53-64 (PMC3247828; doi:10.1186/1751-0147-53-64)
Supplement: Additional file 1 — Outline of study design. Illustration of the relationship between S/P-values relative to the occurrence of the outcome. [file 1751-0147-53-64-S1.PDF]

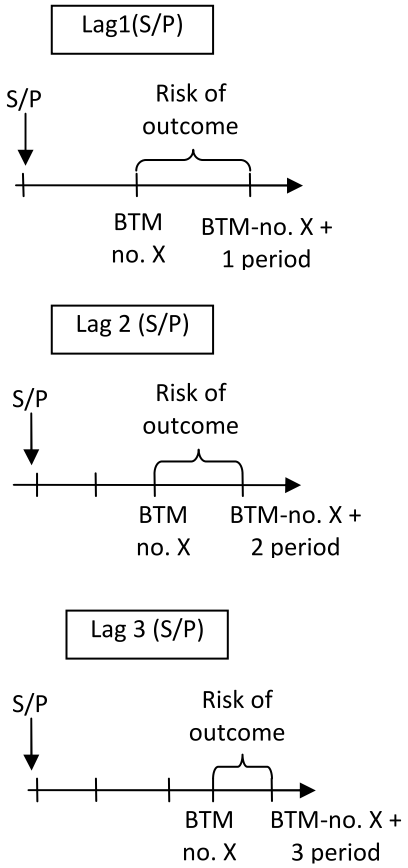

Figure S1. Outline of study design. The S/P-values measured one, two and three periods before the outcome are shown (Lag 1, Lag 2 and Lag 3). S/P-values were measured between 12 periods before and 12 periods after occurrence of the outcome.
